# When standards fall short: The use of discretion and just allocation of long-term care services in Norway

**DOI:** 10.1186/s12913-026-15113-w

**Published:** 2026-07-15

**Authors:** Laila Tingvold, Ragnhild Hellesø, Aud Uhlen Obstfelder, Randi Stokke

**Affiliations:** 1 Centre for Care Research East, Norwegian University of Science and Technology, Teknologiveien 22, Gjøvik, 2815 Norway; 2 Faculty of Medicine, Institute of Health and Society, University of Oslo, Gaustadalléen 21, Oslo, 0349 Norway

**Keywords:** Discretion, Care administrators, Long-term care, Service allocation, Welfare state, ‘Principle of fiduciary’

## Abstract

**Background:**

Care administrators in long-term care (LTC) are ‘gatekeepers’ to the services and handle applications, investigate care needs, and decide if, what and how much services applicants are entitled to receive. Various models of service allocation have been in operation in the Nordic countries since the inception of LTC services in the 1970s. The main drivers of the development of allocation models have been concerns about service inequality among users, quality of care, patient safety, and the overall efficiency within healthcare settings. While care administrators have several laws and guidelines to aid their decision-making, the use of professional discretion is known to be widespread but less investigated. In the context of Norway, this article seeks to explore: What guides care administrators in reaching decisions? How and in which circumstances is the use of discretion needed in making decisions?

**Method:**

Qualitative in-depth interviews with 5 managers in care administration and 13 care administrators in 5 Norwegian municipalities. The material was analysed using thematic analysis.

**Results:**

Two main themes were identified. These are Negotiating standards with discretion and collegial discussion, and Complexities in deciding needs and services.

**Conclusion:**

Care administrators hold significant responsibility in ensuring fair service allocation. Discretion is a necessary and valuable tool that can strengthen the integrity and trustworthiness of care services. However, increased reforms and standardisation place heavy pressure on administrators, limiting their discretion through stricter allocation criteria. This may reduce their ability to act in the best interests of service applicants in line with the fiduciary principle.

**Supplementary Information:**

The online version contains supplementary material available at https://doi.org/10.1186/s12913-026-15113-w.

## Background

The gateway to public long-term care (LTC) services in the Nordic countries has traditionally been regarded as broad and generous, providing care services on a universal principle leading to security for both care recipients and their families [1, 2]. Approximately 400,000 individuals in Norway, representing 7.1% of the population, receive municipal health and care services and over the last two decades, the demand for LTC services has increased among both young and older citizens [3–6]. As the population ages, a continued growth in demand for care services is expected. Public revenues may decrease due to a changing balance between the working-age population and senior citizens. This is expected to challenge the distribution of care services and may lead to a stricter ranking of service recipients and the development of new service arrangements in LTC [7, 8]. Increased standardisation of care and the development of routines have been introduced in health and care services in the Nordic countries over the past decade aiming to ensure efficiency and productivity [9], just allocation, quality of care and patient safety within healthcare settings [9–12]. The aim of this study is to explore how care administrators in Norwegian long-term care (LTC) act as gatekeepers in the allocation of services, with a particular focus on the role of professional discretion within decision-making processes.

In Norway, long-term care (LTC) refers to a range of municipal health and care services provided to individuals who require assistance due to illness, disability, frailty, or age-related functional decline. The services are predominantly publicly funded and allocated according to assessed needs. LTC encompasses home-based services such as home nursing and practical assistance, as well as sheltered housing, rehabilitation services, short-term institutional stays, and long-term nursing home care.

Service allocation is handled at the municipal level, with responsibility distributed across the country’s 357 municipalities, each of which is tasked with organising and delivering public services to its local population. Formally, the allocation of municipal health- and care services follows the procedural rules in the Health and Care Services Act enacted in 2011, and the Patient and User Rights Act that came into force in 1999. According to the Health and Care Services Act, Norwegian municipalities are obliged to offer adequate health- and care services to all citizens who have a need, regardless of age, diagnosis, sex, and ethnicity. The adequacy criterion signifies that municipalities are responsible for ensuring that the services they offer meet recognised professional standards and requirements. This means that services should be safe, ensuring that patients and users are protected from harm to life and health [13]. Hence, when a need for care services is reported, care administrators are obliged to investigate the need and decide within a specified timeframe whether to offer services and which services to offer. If the applicants disagree with the decisions, they are entitled to file a complaint, and the municipality has a three-week deadline to process it. Complaints can also be forwarded to the County Governor. In either case, it shall be possible for the applicants to verify whether they have received fair treatment [14].

Approaches to service allocation have changed since the Nordic LTC services were formed around the 1970s. Initially, Norwegian municipalities assigned the same staff to both assess applications for care services and provide the services granted. This practice was later criticised as inefficient and leading to unequal allocations between service recipients [15, 16]. New approaches to care administration were developed at the end of the 1990s. The model that gained most recognition was the purchaser-provider service (PPS)- model [17]. The model is an approach to LTC service allocation that separates the responsibility for assessing needs and allocating services (the purchaser role) from the delivery of services (the provider role) [18]. The separation was intended to enhance accountability and allowed purchasers to focus on service quality and costs, while providers focused on service delivery. From this point onwards, the role of care administrator became increasingly professionalised, and held by trained health professionals with specialised expertise in areas such as geriatric care, mental health, or rehabilitation. The development of the PPS model was associated with New Public Management (NPM) aiming to improve cost containment, efficiency, organisational flexibility and responsiveness to the service recipients’ needs [19]. The model was accompanied with a free choice of providers in some municipalities and intended to create competition between providers within the community [17, 20] and establish quasi markets [21]. Similar changes in LTC service allocation took place in other Nordic countries [10, 20, 22–26].

In Norway, the PPS model is still the most prevalent in medium-sized municipalities (from 5000 to 20000 inhabitants) and large municipalities (more than 20,000 inhabitants), and about half of Norway’s population lives in a municipality with PPS organisation [27]. However, in recent years, new trends have emerged in service allocation. The divide between purchaser and provider is being modified, which has led to more cooperation between the allocation office and the service providers as well as with the users [27, 28]. For example, it became common for care administrators at allocation offices and home care workers to communicate more frequently to ensure appropriate decisions on type and level of care. Regular meetings are organised to discuss complex cases involving uncertainty in care decisions. In another new approach, the allocation offices have been replaced by ‘early intervention teams’ that combine intensive initial follow-up and tailored approaches to new applicants using a specialised interprofessional team of providers [29, 30]. The team maps new applicants’ needs, resources, and abilities, follows up daily, and tests assistive technology over a few weeks before deciding which and how much care to allocate.

Cutting across the different models and approaches, making decisions on type and volume of care is reported to be both difficult and complicated work [31]. Research has shown variation in levels of care [32], access to care and in care administrators’ assessments and allocation practices [32–36]. Care administrators must make discretionary judgements about what is ‘adequate care’, and the work often implies ranking and comparing the health conditions of various applicants. Ethical dilemmas may emerge when care administrators must prioritise some conditions and care needs ahead of others [15, 31, 37, 38]. In a time of increasing sustainability challenges, less is known about decision-making in the allocation of LTC services. Although previous research has documented variation in service allocation practices and highlighted ethical dilemmas in long-term care administration, less is known about how care administrators navigate increasing standardisation, resource constraints, and competing expectations in their everyday decision-making. In particular, there is limited knowledge about how professional discretion is exercised within contemporary allocation systems and how care administrators balance formal criteria with individual circumstances. By exploring these issues, this study contributes to a deeper understanding of the mechanisms underlying fair and just allocation of long-term care services. Thus, the main purpose of this article is to explore and discuss these topics further. Our research questions are: What guides care administrators in reaching decisions? How and in which circumstances is the use of discretion needed in making decisions?

## Theoretical perspectives

According to the philosopher Harald Grimen, applying discretion implies distinguishing something from something else and judging it [39]. To make good decisions about how to distinguish between different aspects, sound judgement is required. Thus, discretion is always connected to knowledge, reputation, and a sense of judgement. The person who distinguishes between an object, statement, action, or product is attributing or disclaiming characteristics to the area of concern. In other words, a judgement is implicit or explicit based on a ‘yardstick’ (Grimen, 2009, p.2). The judgements can be about statements, conditions, actions, or products. For example, a statement can be true or sincere, an action can be expedient or right, a condition can be normal or desirable, a product good (as in a good knife) or beautiful. True statements are separated from false, wishful conditions from unwanted and a good product from a poor one. But separation is not always easy.

Several conditions can interfere with making a good judgement, as Grimen continues. Firstly, what is separated or considered might not be judged from a single perspective. To decide if an action is fit for purpose, the effects have to be predicted and considered. But the effects may contradict each other, and consequently, they have to be measured against each other before making a decision, claims Grimen. Moreover, any action might also have several unfortunate side effects. All this might complicate a decision on whether an action is fit for purpose. Secondly, the yardstick must be interpreted. For example, what does it mean that something is ‘normal’? Is it a statistical average, such as the average height of people in a country? Or is it something that goes unnoticed because it is taken for granted? It might also be unclear which yardstick should be used [39].

If we engage in these situations, we are presented with unclear circumstances that require discretion. The US philosopher Ronald Dworkin defines discretion as: *‘The concept of discretion is at home in only one sort of context; when someone is in general charged with making decisions subject to standards set by a particular authority’* (Dworkin 1978, p.31). In this sense, Dworkin continues, discretion resembles the hole in a doughnut: there are areas which are left open by a surrounding belt of restriction [51]. For this reason, discretion is a relative term, defined by certain boundaries. In other words, Dworkin views discretion as a space of freedom which is defined within certain restrictions imposed by an authority. What Dworkin refers to as ‘standards’ are those restrictions that define the nature of discretion. These restrictions are set by a specified authority, and they are mandatory for those to whom the authority delegates the role of decision-making [51].

The use of discretion among bureaucrats in welfare states has been discussed by several scholars, and both pros and cons of its use have been addressed. In his seminal work on ‘street level bureaucrats’, Lipsky argues that bureaucrats play a crucial role in the policy implementation process [40]. These individuals, such as social workers and healthcare providers, have significant discretion in how they interpret and apply policies. They are often the ones making key decisions that affect individuals’ lives. Due to the complexity and ambiguity of many existing policies, bureaucrats must navigate uncertain situations and adapt to specific circumstances which require discretion. On top of this, the work situations of bureaucrats are often characterised by high caseloads, limited resources, and the need to make quick decisions [40]. As the judgements made by bureaucrats are not necessarily transparent to the citizens and are operationalised as the final link in the chain of democratic governance, Molander (2012) describes the tension between democracy and discretion through a Rothstein metaphor – the *‘black hole of democracy’* [41–43]. Discretionary powers are delegated and based on trust, expertise, and the expectation that the individual or entity exercising this power will act responsibly and in the best interests (‘principle of fiduciary’) of the beneficiaries [42]. Critics of discretion have, however, opposed the principle. They point towards the risk of inequality and the difficulty of holding persons with discretionary power accountable, as there is always room for arbitrariness [44]. Thus, by interfering with citizens private sphere, discretion threatens to undermine the rule of law [45]. A legal concern which also raised is when persons holding discretionary power do not implement the law and policies as intended [43]. This may lead to conflict with the principle of equal treatment and democratic governance.

Another source of criticism stems from research on the psychology of judgement and decision-making [42]. Since finding an optimal solution is sometimes impossible or impractical, heuristic approaches (cognitive simplifying techniques) can be used to speed up the process of finding a satisfactory solution [46]. The heuristics reduce complex problems to simpler cognitive tasks, and lead to efficiency in decision-making. The assessments obtained by using heuristics are often good enough [42], but may also lead to systematic biases [47].

We will apply these theoretical perspectives to analyse how care administrators allocate services, and return to them in the discussion to contextualise our results.

## Method

### Research design and participants

We conducted a qualitative study using semi-structured interviews with employees in service allocation units in five municipalities. To capture diverse organisational contexts, we selected municipalities based on variation in organisational models, sizes (small: under 5,000 inhabitants; medium to large: 15,000–120,000 inhabitants) and geographical locations across different regions of Norway. Managers in service allocation units were approached to participate and to suggest three to five care administrators with direct experience of service allocation across different care contexts such as home care, nursing homes, or sheltered housing. The care administrators gave their consent to participate. Details about the participants are given in Table 1 below.

**Table 1:** Study participants

Participant	Municipality	Age	Education	Years in LTC	Position	Years in position	No. interviews
1	A	53	Nurse	12	Head of allocation office	4	2
2	A	41	Social worker	15	Care administrator	3	1
3	A	60	Social worker	8	Care administrator	2	1
4	A	37	Nurse	13	Care administrator	2	1
5	A	41	Nurse	14	Care administrator	5	1
6	B	62	Nurse	32	Head of allocation office	20	2
7	B	33	Social worker	13	Care administrator	2	1
8	B	61	Nurse	31	Care administrator	14	1
9	B	48	Nurse	18	Care administrator	2	1
10	B	51	Nurse	11	Care administrator	4	1
11	B	55	Nurse	24	Care administrator	2	1
12	C	53	Nurse	7	Head of allocation office	2	2
13	C	32	Nurse	9	Care administrator	1	1
14	C	39	Nurse	8	Care administrator	2	1
15	C	42	Social worker	10	Care administrator	1	1
16a/16b	D	47/51	Nurse/Nurse	10/15	Head of allocation office/care administrator	8/2	1
17	E	34	Social work	11	Head of allocation group	1	1

### Data collection

The first author conducted interviews between May 2022 and July 2023. An interview guide was drafted and informed by reports and research papers addressing care administration in LTC services. The interview guide was discussed among the co-authors and pilot-tested by the first author with a manager from a municipal service allocation unit who was not included in the study sample. The pilot interview was used to assess the relevance and clarity of the questions, as well as the overall sequencing of topics. Based on the pilot, minor revisions were made to the wording of several questions, and additional questions were included regarding the laws, guidelines, and other decision-support tools used by care administrators in their daily allocation work. The final interview guide addressed participants’ background (role, education, work experience); the organisation and authority of the allocation office (structure, decision-making powers, collaboration); daily work (tasks, workload, service demand); decision-making processes (simple versus complex cases, application of laws and standards, documentation, follow-up); allocation of welfare technology (types offered, criteria, challenges); changes in services and collaboration with civil society (service phase-out, “care staircase” approach); and concluded with space for additional reflections. The five initial interviews with managers in care allocation offices were conducted over Teams. The follow-up interviews with care administrators were conducted physically in the municipalities. The interviews lasted 40 to 95 min, depending on how much information the participant wished to share. All interviews took place at the offices of the care administrators and were audio-recorded and transcribed verbatim.

### Participants

All participants in this study were women. They were between 32 and 62 years of age and had extensive experience in LTC varying from 9 to 32 years. As shown in Table 1, all participants were educated within health sciences and were mainly nurses. They all had additional education within management or specialist competence within various fields such as rehabilitation, geriatric nursing, psychiatry, addiction, habilitation.

At the time of the interview, 11 out of 18 participants had limited experience (3 years or less) in their actual job as care administrators. Three of the five municipalities had care administrators on long-term sick leave and two had had vacant job positions for a lengthy period of time with no qualified candidates to fill the positions.

### Analysis

A thematic analysis (TA) inspired by Braun and Clarke [48–50] was applied. The analysis was conducted manually through an iterative and reflexive process. In line with the first phase of the TA approach – *familiarising yourself with the data* [48], we began the interpretive process by reading the transcribed interview texts. The first author shared a selection of transcribed interviews and her notes from the interviews and organised the material into broader themes. The co-authors formulated keywords and reflections from their readings. Four research team meetings were organised among the authors in which we shared and discussed our initial ideas and preliminary understanding of what the texts conveyed. When familiar with the data, we moved on to *generating initial codes* [48, 50]. To identify featurer of the data and organise them into meaningful groups, the first author performed the initial coding. Having identified our initial codes, we then searched for patterns related to our research aim. By doing so, we initiated the process of *generating themes* [48, 50], in which we focused attention on identifying interesting items in the data that we believed could form the basis of repeated patterns, i.e. potential themes across the data set.

In the process of *reviewing themes* [48–50], we continued to explore the coded extracts of each theme to evaluate whether they formed part of a coherent pattern. We then moved on to *defining and naming themes* [48]. In this process, we attempted to refine the themes so as to capture their essence, aiming to describe our interpretations of the care administrators’ perspectives as clearly as possible. Consequently, it was possible for us to formulate the content of each theme in a few summary sentences. These concise formulations were then used to develop the theme titles – from working titles to final titles of the themes. An example from phases in the analysis process is enclosed in Table 2 below. All authors are experienced qualitative researchers with expertise in long-term care (LTC) health services research, and three authors have in-depth insight into the specific study theme. Throughout the analysis, interpretations and emerging themes were discussed among the authors to challenge assumptions and enhance reflexivity. Differences in interpretation among the authors were discussed until a shared understanding of the themes was reached. Alternative thematic constructions were considered and compared throughout the analytical process.

**Table 2:** Example from phases in the analysis process

**Familiarising ourselves with the data – data extract** *‘…and for making decisions on services, it’s not clear either … how much [services] should be allocated. So, there is an extreme amount of individual judgment here! Because … for two apparently similar applications and similar individuals, when you start talking to them and see the specific conditions they live under or … what networks they have around them, the decisions can be completely different.*		
**Generating initial codes** a. Individual judgement b. Conditions and networks surrounding service applicants are different		
**Generating themes** −Applicants’ house and living conditions are different and impact decisions −Consideration of whether there are family members or other support networks −Consideration of long−term effect of a decision −Consideration of distance (centre and periphery) may impact	**Reviewing themes** Several conditions had to be judged by discretion and by collegial discussions in addition to the health and care need. This often implied consideration of the living conditions of the service applicant, if they had family or networks to support them in addition to the care services. Judgements of the long−term effect of an allocation was also considered	**Defining and naming themes** −Negotiating standards with discretion and collegial discussions

### Ethics

According to Norwegian legislation on research in medicine and health (Health Research Act), ethical approval was not needed. The ethics of the study were guided by the World Medical Association’s Declaration of Helsinki. Approvals on the protection of privacy were obtained from the Norwegian Centre for Research Data (NSD, now SIKT), project number [474050] and the municipality involved. All the methods and procedures were carried out in accordance with their guidelines and regulations. The first author had initial meetings with the management in the included municipalities and presented the study. The managers of the allocation units gave their consent for their unit to participate in the study before the recruitment of the care administrators took place. Afterwards, the managers of the allocation units in the municipalities informed and asked the staff for their participation. All participants gave written informed consent to participate in the study. The participants were informed of their right to withdraw from the interview without stating a reason, and the data were managed with respect to the anonymity and confidentiality of all participants. All names and data about the municipalities that could lead to identification were omitted in the transcriptions.

## Results

We identified two main themes related to our research questions: negotiating standards with discretion and collegial discussions, which comprised three sub-themes; and complexities in deciding needs and services, which comprised two sub-themes.

### Negotiating standards with discretion and collegial discussions

#### Formal frameworks guiding allocation decisions

The care administrators referred to several tools that guided allocation of services. Four general laws guided the overall service allocation: Health and Care Services, Act Patient and User Rights Act, Health Personnel Act, and Specialised Health Services Act. The direct formulations used in the Acts were seldom studied by the care administrators as they were seen as too general for daily service allocation. However, general formulations of the formal rights to receive care services were inserted as standard text in all letters sent to service applicants. In making decisions, the care administrators considered the three national criteria for prioritising health and care services introduced by the Norwegian government in 2020. The criteria are: ‘benefit’, giving priority to allocation measures with greater health gains; ‘resource use’, favouring allocation requiring fewer resources; and ‘severity’, prioritising more serious conditions (Ministry of Health and Care Services, 2021). A guide developed by The Norwegian Directorate of Health in 2017 was also in use to help ensure that the right services were provided at the right time and in sufficient scope. The guide was designed as an educational tool for training caseworkers and managers in best practice procedures.

#### Collegial discussions as support for discretionary judgement

The participant expressed they had developed their own municipal service standards to better specify the conditions and criteria, that grant access to the different types of services. These standards were applied to clarify who should receive services. They also often consulted their colleagues to obtain support for their interpretations of standards and decisions in daily practice. The colleagues they consulted were either other care administrators at the same allocation office or their managers.

For example, in one municipality, a care administrator described a case concerning a woman in her mid-nineties who had applied for a place in a nursing home for the second time. The woman was mentally alert but had poor physical health with painful wounds which made it hard for her to move around in her house. She spent most of the days in bed and the home care services attended to her four times a day to tend her wounds. Staff from the home care services had notified the care administrator that the elderly woman suffered considerable pain, and she had also repeatedly stated a wish to die. She had one distant relative who visited a few times a month, and apart from that she had no visits other than from the home care staff. The care administrator stated that she had rejected the woman’s application for a place in a nursing home for the second time and referred to the service standard in the municipality to justify the decision:

*In our [municipal] service standard*,* we keep the spots in the nursing homes for people needing help around the clock and for elderly with dementia. All other cases we refer to home care services.*

The care manager explained that she felt quite certain about her decision but had still chosen to bring up the case in weekly meeting with her colleagues. When the care administrator was asked by her colleagues why she brought up the case for discussion, she stated that the old age of the woman and her known depression were disturbing factors that made her question whether the dignity of the patient was being respected and if the services offered were within the legal framework. When explaining her decision, she claimed that the elderly woman would have benefitted from getting more social contact, but that *‘people’s loneliness was not the responsibility of the municipal care services’.*

This case illustrates that while municipal service standards provide clear guidelines for determining eligibility for care services, they cannot fully account for the complex ethical considerations involved in individual cases. The care administrator’s decision, though aligned with policy, was accompanied by uncertainty about whether the dignity and emotional needs of the patient were being met. By choosing to bring the case to her weekly meeting, the administrator demonstrated that collegial support functions as more than a procedural formality — it is a vital forum for critical reflection, moral reassurance, and the exchange of perspectives that can illuminate dimensions of care otherwise overlooked.

In this instance, the colleagues’ probing questions helped frame the woman’s situation not only in terms of medical necessity but also in terms of human dignity, mental health, and social isolation. Collegial dialogue thus provided the administrator with a safe space to confront the tension between policy constraints and ethical responsibility, reducing the risk of moral distress and enhancing the quality of the decision. This underscores that in health and care services, collegial support is not merely a supportive resource for staff, it is an essential part of safeguarding ethical standards and ensuring that care decisions remain person-centred, even under restrictive regulations.

#### When discretion overrides standards

In another case, a care administrator decided to deviate from the municipal service standard. The case concerned an elderly man who had a considerable need for care services. Both frailty and cognitive decline rendered him dependent on help and care from his spouse. Healthcare staff often observed spouses on the brink of exhaustion, and alerted the care administrators and encouraged the spouses to apply for a short term stay in the municipal rehabilitation unit for their partner. The care administrators recognised that in most of these cases, the potential for rehabilitation was totally absent, but a short-term stay at such a location could reduce the care burden on the spouse and help her or him regain energy. The municipal service standard specified that the places in the rehabilitation unit were earmarked for users with rehabilitation potential. The care administrators asserted, however, that in these cases they had to prioritise the long-term effect and keep the spouse healthy by helping to make them take care of themselves as well as their partner. A service administrator claimed that this could postpone the supply of care services and stated:

*Sometimes we must think of what the long-term effect of a decision is. Is it a preventive measure? Then we use discretion. So*,* if they [service recipients] do not meet the conditions now*,* we can still approve because we see that it has a long-term effect which means that they are able to keep their level of functioning at a stable level.*

The care administrators argued that overriding the municipal service standards was favourable for everybody compared to getting another new client (the spouse) permanently dependent on care services as well.

Returning to the general laws, care administrators claimed that these were useless when it came to deciding which types of services clients should be offered, or how much help to offer. Standardisation and care pathways were not helpful either as the applications for services for similar needs e.g. care related to cancer or hip fractures, could end up with quite different decisions. One care administrator stated:

*…and for making decisions on services*,* it’s not clear either … how much [services] should be allocated. So*,* there is an extreme amount of individual judgment here! Because … for two apparently similar applications and similar individuals*,* when you start talking to them and see the specific conditions they live under or … what networks they have around them*,* the decisions can be completely different.*

For example, two service applicants might have the same diagnosis (e.g. hip fracture) but completely different lives. One might own a comfortable house adapted to a person with mobility challenges, while another might live in a run-down house, with an upstairs bathroom and overall poor maintenance. One service applicant might have several relatives or good supportive neighbours to reach out to, while the other might lack personal relations. One might live in quite a central area and have easy access to a service centre, grocery stores, pharmacy etc., while the other might live far away in the countryside.

This case above shows how care administrators sometimes deviate from municipal service standards when rigid adherence would undermine both patient welfare and long-term system sustainability. Although rehabilitation unit places were formally reserved for individuals with rehabilitation potential, the administrator allocated such a place to an elderly man whose needs were primarily custodial. The decision was not based on his recovery prospects, but on the need to relieve his exhausted spouse and prevent her from becoming a future care recipient. In doing so, the administrator weighed several intertwined considerations: the immediate (non)availability of alternative respite options, the ethical responsibility to protect the caregiver’s health, and the strategic aim of reducing future care costs by postponing or avoiding additional long-term service commitments. This illustrates that resource allocation decisions in municipal care are rarely one-dimensional; they require balancing individual needs, policy frameworks, ethical duties, and systemic constraints, often in situations where no option fully satisfies all priorities.

In summing up, care administrators allocate services using general laws, national prioritisation criteria, municipal service standards, and professional discretion, often consulting colleagues to navigate ethical dilemmas and case-specific complexities. While standards provide structure, administrators sometimes deviate from them to address broader welfare concerns, balance long-term resource use, and safeguard both patient dignity and caregiver well-being.

### Complexities in deciding needs and services

#### Assessing and defining care needs

Almost all applications for care services contained an element of using discretion in decision-making. The care administrators differentiated between cases requiring substantial discretion where the allocation process was complex and time-consuming, and cases that were quite straightforward and fast to resolve.

Disagreements about judging and defining the ‘need’ for care services were repeatedly mentioned as an issue that was often difficult to handle, especially if it concerned new applicants about whom they had no previous information. A typical example was when a spouse, son or daughter applied for services on behalf of a partner or a parent. The application might state that the person had a large unmet care need or was becoming confused and unable to live alone. The care administrators found such information useful and correct in many cases, but they had also found service applicants in completely different conditions, better or worse. A care administrator explained that discussions could arise between care administrators and service applicants, between care administrators, but also between the family members of the service applicant regarding what the ‘need’ consisted of:

*There can be a large degree of disagreement between children* [of a service applicant] *as well. The one child who lives close to his relative experiences that their level of functioning is much worse than those [siblings] who live far away and who will then invoke the right of the mother to decide for herself*,* for example. And then … she…the mother… is observed … in … a fine blouse and a handbag and … just a pantyhose*,* for example. Then it is obvious that something is not working.*

In such cases, the care administrators had to spend time assessing, judging and clarifying the need for help, what services where required and how quickly care services should be provided. They looked for signs of cognitive decline or uncritical behaviour in the service applicant and evaluated any incoming information about the service applicant continuously to judge if the municipality was operating within the ‘accountability’ criteria as formalised in the law.

Much time could pass, especially if there was a need to clarify the service applicant’s capacity to consent. The general practitioner was contacted to examine this. In this process, it could be difficult for the care administrators if the service applicant did not want to communicate with them and refused to open the door to healthcare staff. Visits by healthcare staff deliverering medication and meals or practical assistance often provided valuable information about the condition of the service applicant and helped determine need and urgency.

Cases demanded less discretion if the need for care was indisputably known, and if the care service had a clear aim and timeframe. Examples of such situations were persons needing home care services to tend a wound until it healed or granting an old person living alone a place in daycare centre a few times a week to ensure healthy meals. More complex judgements had to be made if there were no openings at the day centre or if several service applicants were competing for one available spot.

Sometimes, care administrators thought the service applicants requested far more care services than they needed and had too high expectations as to what they could receive. The care administrators were careful not to offer ‘too much’ and claimed it would not benefit the person. They spent time assessing service applicants’ physical capacity prior to making decisions and conducted home visits to get to know new applicants. One care administrator explained that she had recently handled an application for practical assistance. During the home visit the administrator found that the applicant was able to bend and touch the floor and to do some cleaning herself. The care administrator decided not to offer as much help as the applicant had wished for, and instead included her in a home rehabilitation program. The care administrator said:

*…many of the applicants can do more than they think! So*,* then we must figure it out. And then we’ll go in and compensate for what they can’t do then. We… put in some intensive home rehabilitation. We don’t want to put them in a situation where we nurture them passively. So*,* we would like to start rehabilitation at home and … get them back on their feet quickly and enable them to fend for themselves with simple measures.*

This example shows that the care administrator’s judgement and decision relied on two considerations; first the belief that it would be better for service applicants to stay active and to master daily life and functions as long as they could, and secondly that these decisions were in line with the policy of keeping the service recipient at the lowest possible care level, saving costs in an already pressed municipal economic situation.

#### Complexity requires extensive experience and expertise

To make decisions in this complex terrain, the managers stated that during hiring processes they looked for qualified health professionals. They sought staff with substantial work experience within health and care services (home care, nursing homes, hospitals) and with the robustness required for the demanding role of decision-making. The manager of an allocation office stated:

*You need someone who can stand in a storm*,* someone who is robust… who can make decisions and who can… manage to speak up when it’s enough. Manage to ask for help… and they* [care administrators] *must be interested in… law and economics… we also have the economic responsibility here*,* and they should also know that when they make a decision*,* they generate costs for the municipality and use our budget.*

Other qualifications mentioned to be required in care administrators were good knowledge of the local community, communications skills, and ability to handle short timelines and cooperate very well in a team. When a care manager in the interview had listed all qualifications and qualities she was looking for in a new employee, she exclaimed that being a care administrator: *‘…is not a job for exhausted nurses’.* Care administration was generally addressed as hard work among the study participants.

In summing up, care administrators face varying levels of complexity in assessing care needs, from straightforward cases to those involving conflicting information, family disagreements, or uncertain capacity to consent. They rely on professional judgment, home visits, and medical input to determine the urgency and scope of services, aiming to keep recipients as independent as possible while managing limited resources. The role demands robust health professionals with strong legal, economic, and communication skills, able to make difficult decisions under pressure.

## Discussion

This study provides insights into the allocation processes of long-term care services at a time with emerging sustainability challenges, limited resources, and budget austerity. In their daily work, the care administrators claimed to comply with formal laws and policy guidelines as well as with service standards developed in the individual municipalities. The use of discretion and collegial discussions was, however, often pivotal for making decisions on type and level of services. Below we will discuss the use of discretion as well as the ‘principle of fiduciary’ in relation to the theoretical perspectives.

### Discretion – the world beyond standards

In the theoretical background in this paper, we outlined Grimen’s (2009) problematisation of the ‘yardstick’, emphasising that good judgement is needed in distinguishing something from something else [39]. In our empirical material we have documented how care administrators apply Grimens concept in practice in striving to define ‘need’ and judge which service is ‘adequate’. This process could be highly complex. Care administrators sometimes became entangled in several opinions and spent considerable time identifying and judging what the actual ‘need’ was, considering various perspectives before making decisions.

The process of allocating care was not only judgements about the service applicant’s health condition, but also judgements about their surroundings. For example, was the service recipients house ‘safe enough’ (for an elderly person with frailty or cognitive impairment to live alone in, or if any relatives lived close by), ‘functional enough’ (could the person access the bedroom and bathroom by themselves), and was the house and person living there ‘clean enough’ (kept a clean house, removed trash and was able to take care of personal hygiene)? These were some conditions that frequently interplayed with ‘need’. In addition, judgement was required in considering which services would be ‘adequate’ or ‘enough’ or ‘beneficial’ for the service applicant, and the availability of these in the municipality. This illustrates a complex terrain of considerations to be taken, and the need to use discretion and collegial discussions was imperative. It supports the view that using discretion is necessary when factors are unclear or even conflicting [40, 51]. Laws and standards constitute a general background framework but the use of discretion as well as collegial discussion was important tools for the care administrators in their daily work. In discussing the nature of care and discerning judgement, Alvsvåg and Martinsen claim that good judgement and discretion form a connection between a unique situation and knowledge, norms and laws [52]. The use of discretion also creates links between different sources of knowledge and professional competence. Our study supports these considerations.

In the introduction to this paper, we presented the different approaches and models directing the allocation of LTC services over the years. The models reflect changing views on how close or distant the care administrator should be from the service applicant to make ‘just’ or ‘equal’ decisions and create the most efficient allocation system. In the purchase-provider service (PPS) model, the gap between purchaser and provider was argued to be important to safeguard equal allocations. However, studies have shown that direct and frequent communication and cooperation between those contracting and providing the services is sorely required. Care administrators need up-to-date information and knowledge of the service recipient to make sound judgements and decisions [18, 53]. This becomes especially important as the health conditions of many service recipients (especially multimorbid elderly) may change rapidly, requiring swift adjustments in services [54–56]. Our study confirms the importance of care administrators being updated and informed about the health and life changes of service applicants. This information is crucial to make timely and sound decisions with discretion. Our findings illustrate Grimen’s argument that professional judgement requires distinguishing between competing considerations rather than simply applying predefined criteria. Although municipal standards and legal frameworks provide important guidance, care administrators frequently encountered situations in which several relevant considerations had to be weighed against one another. The extensive use of collegial discussions further suggests that discretionary judgement is not solely an individual activity but is often developed and validated through collective professional reflection.

### Between expectations and realities: Principle of fiduciary

Discretionary powers are delegated (to care administrators) and based on trust and expertise and the ‘principle of fiduciary’; the expectation that the individuals exercising this power will act responsibly and in the best interest of the service recipient [42]. Care administrators in this study tried to adapt to a situation with limited resources in terms of time, personnel, and budget constraints. They complied with the current policy trend of being less ‘generous’ than before, e.g. not allocating services for tasks that the applicant could perform without help [57, 58]. Dilemmas occurred, however, when the care administrators saw that the services they could offer home dwelling elderly, were insufficient and rendered elderly people lonely or striving. In addition, care administrators had to handle criticism from the service recipient’s family who often demanded more or different help for a parent or a spouse. Earlier studies have addressed the issue of care administrators finding that they are pressured by increasing expectations from the family members of service recipients [59], as well as increased expectations from the care services management to always allocate at the lowest possible level [36]. As also illustrated by Lipsky, balancing these conflicting demands from both the public and higher-level authorities are challenging [40]. According to Lipsky, the frontline workers holding the direct responsibility for implementing policy are constrained by organisational rules and resources. Decisions might be taken under ambiguous and challenging circumstances, which could lead to tension between adhering to formal procedures and responding effectively to the needs of the individuals they serve. This demanding situation highlights the need for care administrators to have targeted competence to perform their work and manage the pressure. They need professional healthcare education, long-term work experience in the sector, local knowledge, and the managers responsible for hiring who seek robust personality types. Possessing these qualifications might ease the handling of conflicting demands and complaints.

The use of the ‘principle of fiduciary’ among care administrators can also be discussed in light of current health and care reforms. A prime focus in policy and reforms today is ‘active ageing’ that emphasises enablement and rehabilitation to encourage self-sufficiency and postpone the need for care services [60–62]. The current trend in municipalities is to establish specialised teams with staff focusing solely on rehabilitation [63] and working to ensure that elderly people can live longer in their own homes in accordance with the ‘Live safely at Home’ reform [64]. Efforts are made to try to rehabilitate and train users in self-mastery prior to making any decisions on allocating care services [65]. The participants in our study were responsible and largely loyal to policy directives but also encountered dilemmas such as allocating services within the ‘accountability’ criterion knowing that the service applicant (or the spouse) would have been better off with more help or other services. In our material we notice a trend to rank physical healthcare needs ahead of psychological and emotional needs. Such ordering of needs runs counter to the professional obligation to provide holistic care or whole person care [66–68]. Even if the allocation is within one standard or ‘yardstick’ (e.g. municipal standards), it might contradict other standards at work. For example, the professional ethical guidelines for nurses state that *‘…The nurse takes care of individual patients’ needs for holistic care*,* and supports human resources*,* coping*,* hope and courage to live’* [69]. This reflects a paradox and tightrope, forcing care administrators to balance policy on the one hand and the opportunity to use discretion on the other. Under these circumstances, it is questionable whether the allocations made are in the best interest of the service applicants. The scope to act in the best interests of the service recipients seems to be compromised or narrowed and challenged.

Another aspect of knowing what would serve the service recipients best is that situations can be highly unpredictable, making it difficult to make informed decision. A situation can have so many possible outcomes and may be influenced by a number of unknown factors. Care administrators also speculated about the long-term effects of their decisions, e.g. if allocating increased home care services would lead to improvement in the lives of service recipients or passivity which eventually would be negative. Nevertheless, estimating and predicting these side effects was presented as hard and highly challenging.

Lipsky’s work highlights the importance of understanding the role and experiences of the frontline workers in the effective delivery of public services and the impact of their decisions on the recipients of those services [40]. In a time of increased standardisation and reforms pressing for less generous services, we question how much leeway is left for discretion. In the future, care administrators will handle applications from service applicants living with more complex health and care needs in their individual homes. If the pressure on care administrators is such that it jeopardises their ability to use discretion and the principle of fiduciary, we risk being left with standards and less space for individual solutions.

Also, our material shows that care administrations must have many qualifications resembling the forces of ‘super women’ to handle the job. They often had to make decisions under unclear situations and for vulnerable service applicants with unpredictable health conditions.

The findings also illustrate Dworkin’s description of discretion as operating within a framework of rules and restrictions. Care administrators exercised discretion not in the absence of standards, but within the space left open by legislation, municipal criteria, and organisational constraints. At the same time, increasing standardisation and resource pressures appeared to narrow this discretionary space, potentially limiting their ability to act in accordance with the fiduciary principle and tailor decisions to the individual circumstances.

## Conclusion

Care administrators handle an increasing number of applications from a diverse group of citizens of all ages with different reasons for needing care. The care administrators are familiar with policy guidelines and standardised municipal criteria for accessing care services and strive to comply with them. As we have seen in this study, a large part of the work related to service allocation is, however based on the use of discretion. Contrary to the critics of discretion who point to the risk of inequality or that other policies than intended are being implemented, we argue that discretion is a necessary tool in safeguarding just allocations. Service applicants have different situations, and the use of discretion may help to tailor the services individually. However, we are concerned about the opportunity and leeway for applying discretion in the future. Reforms and standardisations may limit the freedom entrusted to care managers by introducing stricter criteria. For example, when spaces in nursing homes are earmarked for elderly needing help around the clock, care administrators will be compelled to allocate services to a large group of marginal and frail elderly people living at home. Such reductions in the care portfolio is likely to violate the ‘principle of fiduciary’ and compromise or limit care administrators’ right and ability to use discretion.

We have also seen that care administrators handle many and sometimes conflicting expectations. In line with national policy, they are to allocate adequate care at the lowest possible level. At the same time, they must be sensitive to the needs of service applicants and handle demands and complaints from service recipients and their families. To cope with the pressure, care administrators need solid competence and qualifications within health and care as well as personal strength and communication skills.

### Trustworthiness and limitations

The study’s trustworthiness is supported by several factors. Credibility is strengthened through rich, in-depth interviews with managers and care administrators, combined with participants’ extensive experience, and by the use of collaborative thematic analysis to reduce bias. Transferability is enhanced by detailed descriptions of the Norwegian LTC context, organisational models, and participant backgrounds, enabling readers to assess applicability to other settings. Dependability is ensured through transparent documentation of sampling, data collection, and analysis, alongside the use of a tested interview guide and consistent procedures. Confirmability is supported by grounding interpretations in participants’ own words and theoretical perspectives, with collaborative analysis ensuring reflexivity and transparency.

This study has limitations that should be acknowledged. The study is based on interview accounts rather than observations of allocation meetings or actual decision-making processes. Participants described how they make decisions and use discretion, but there may be differences between reported practices and decisions enacted in real-life situations. Observational studies could provide additional insight into how discretion is exercised in practice. The findings are based on interviews with care administrators from only five Norwegian municipalities, which may limit the generalisability of the results to other regions or countries. No comparisons are made related to type of service allocation model among the municipalities. As the study relied on self-reported accounts, there is a possibility of recall bias or social desirability bias influencing the responses. A limitation of this study is that the findings were not disaggregated by managerial and care administrator roles. Because participants were recruited through managerial access procedures, some managers who also performed allocation tasks were included. While interviews focused on participants’ own practices, managers may hold perspectives shaped by their responsibility for service quality and efficiency, potentially leading them to emphasise adherence to formal guidelines over discretion. This possible bias should be considered when interpreting the findings. Furthermore, the perspectives of service recipients and their families were not included, which might have provided a more balanced and comprehensive understanding of the allocation process. Finally, the findings are shaped by the specific policy and organisational context in Norway and may not be directly transferable to other welfare systems.

## Supplementary Information

Below is the link to the electronic supplementary material.

Supplementary Material 1

## Data Availability

The datasets generated and/or analysed during the current study are not publicly available due to the protection of the participants and the municipalities anonymity that was granted them prior to the interviews. Anonymised short summaries of the interviews can however be made are available from the corresponding author on reasonable request.
