# Supplementary material for: When standards fall short: The use of discretion and just allocation of long-term care services in Norway
Source: BMC Health Serv Res. 2026 Jul 15;26:1382. doi: 10.1186/s12913-026-15113-w (PMC13660045; doi:10.1186/s12913-026-15113-w)
Supplement: Supplementary file 1 [file 12913_2026_15113_MOESM1_ESM.docx]

**Interview guide allocation office (managers, employees)**

Personal information

Position today/Education/Age/Work experience:

About the establishment, organization, and authority of the allocation office

1. What label is used for this allocation office? (a. Purchase- provider), b. committee, c. (variations of) the trust model, d. other, specify?)
2. How long have you had this organization, and what did you have previously? Why were changes made?
3. Are there plans for changes in the organization of the allocation office soon? Any justifications for this?
4. If the allocation office is divided into several zones or sections, can you tell me about the tasks each one has?
5. Which groups and types of services are you particularly familiar with allocating? (Children, substance abuse/psychiatry, intellectual disabilities, elderly, refugees, home-based services, institutional services, etc.)
6. What decision-making authority does your allocation office have? (Autonomous, dependent on others)
7. If you don't have/little decision-making authority – who do you need to consult with? Who is the real decision-maker? Can you tell me about this collaboration and give examples of some cases.
8. What other (if any) actors do you collaborate with in the work of allocating services?

A workday with service allocation

9.	Tell me about a typical workday you have with service allocation. What works well and what doesn't?

10. How was the last shift or the last week? Anything particularly positive/negative? Special events?
11. How do you experience the demand for services? (Stable, increasing, decreasing)
12. Have there been noticeable changes in the last year/six months?
13. What impact has the pandemic had on the allocation of services?

Decisions

14. What decisions did you make yesterday? Were any of these particularly challenging?

15. Describe the process of an easy/uncomplicated decision?
16. Describe the process of a complicated decision?
17. What laws, regulations, and materials do you rely on when allocating services?
18. Which actors are involved in cooperation regarding a decision? Are decisions made individually by care administrators, if so, what cooperation do you have internally at the allocation office?
19. What is the process? What is on paper? What is digital? What is the flow of the process from the incoming case to the decision being made?
20. Are there any challenges in the digital tools you use? Can you tell me about these?
21. When a service is approved, how is the need for these services followed up? Are there evaluation points? Does it happen that services are reduced and terminated, increased? What processes are involved in changing decisions about services?

Welfare technology

22. What types of welfare technology do you offer in the municipality?

23. What determines whether a user should receive welfare technology or other services? Who makes the decision? Who do you collaborate with? (relatives, users, the service provided previously, general practitioner, hospital, home care, occupational therapist, physiotherapist)
24. Do you see challenges with the allocation of welfare technology? How do you follow up with the user? Do you review how the solution works? If so, what challenges exist, and how are they solved?
25. Do you have examples of how welfare technology has changed the offer of services or affected the service in various ways?

Civil society and phasing out services

26. Do you collaborate with voluntary organizations or agencies that offer low-threshold services for individuals with (emerging) health and care needs?
27. Health and care services are developing, new services are introduced, and others phased out. Do you have examples of services that have been phased out in recent years?
27. Does the allocation office use the care ladder when assessing service recipients?
28. Anything we haven't asked about that could be useful for us to know?

Thank you -
